# Improvement of Indoor Residual Spraying and Long‐Lasting Insecticidal Net services through structured monitoring and supervision as part of the Malaria Elimination Demonstration Project in Mandla, Madhya Pradesh

**DOI:** 10.1186/s12936-021-03639-9

**Published:** 2021-02-18

**Authors:** Ashok K. Mishra, Sekh Nisar, Harsh Rajvanshi, Praveen K. Bharti, Kalyan B. Saha, Man Mohan Shukla, Ravendra K. Sharma, Himanshu Jayswar, Aparup Das, Harpreet Kaur, Suman L. Wattal, Altaf A. Lal

**Affiliations:** 1 Indian Council of Medical Research - National Institute of Research in Tribal Health (ICMR- NIRTH), Jabalpur, Madhya Pradesh India; 2 Malaria Elimination Demonstration Project (MEDP), Mandla, Madhya Pradesh India; 3 Directorate of Health Services, Government of Madhya Pradesh, Bhopal, Madhya Pradesh India; 4 Indian Council of Medical Research, Department of Health Research, Ministry of Health and Family Welfare, New Delhi, India; 5 National Vector Borne Disease Control Programme, Ministry of Health and Family Welfare, New Delhi, India; 6 Foundation for Disease Elimination and Control of India, Mumbai, Maharashtra India

## Abstract

**Background:**

The Government of Madhya Pradesh employed Indoor Residual Spraying (IRS) with alpha-cypermethrin synthetic pyrethroids in sub-centres with Annual Parasite Incidence (API) from 2 to 4.99. In sub-centres with API more than 5, Long-Lasting Insecticidal Nets (LLINs) were distributed. At the request of the State Government, the Malaria Elimination Demonstration Project (MEDP) staff observed and provided support to both IRS and LLINs campaigns. In the year 2017, the study team monitored only the IRS campaigns, however, in the year 2018, the supportive supervision was provided to the IRS campaign teams along with post-distribution monitoring of the LLINs.

**Methods:**

The study was carried out during IRS spraying using a pre-tested, closed-ended monitoring checklist which consisted of two parts- observations of spraying team and observation of sprayed houses. For LLINs, a sample of the households that received the bed nets was taken for the study. For IRS, the spraying teams were monitored for quality and technique for a total of 159 times in 2017 and 183 times in the year 2018, respectively. For post spraying observations, a total of 1261 and 1791 households were observed in the years 2017 and 2018, respectively. The use of LLINs was observed in 5 % of the households in 2018 and 2020, which is about 2,000 houses in each survey where each house received about 2.5 LLINs per household. The results of surveys were compared to assess impact of supportive supervision and monitoring.

**Results:**

Significant improvement was noted after supportive supervision in year 2018 in various aspects of spraying. Preparedness of spraying, such as advance information to villagers, presence of equipment and records improved by up to 70 %. The methodology of spraying preparation improved from 50 to 90 %, spraying technique improved from 54 to 80 %, and proper use equipment during spraying improved from 51 to 92 %. After eight months post distribution of the LLINs in 2019, improvement was seen in regular usage of LLINs by 28 %. It was found that on-spot demonstrations during distribution and carrying of LLINs when sleeping outside homes increased by 56 %. Results of IEC campaigns revealed the reduction in adverse effects by 64 % and increase in awareness by 97 %.

**Conclusions:**

Effective supervision improved the quality of IRS and usage of LLINs in the study area. Based on these results, continued training and monitoring of staff that is deployed to spraying houses and distribute bed nets was suggested. The study also revealed that proper IEC/BCC drives help increase community acceptance of vector control measures and their rational usage.

## Background

Malaria is the most common vector-borne infectious disease of tropical and subtropical regions [1]. In India, Madhya Pradesh (M.P.) is the fourth most malaria endemic state, contributing about 7 % of total malaria cases [2]. Vector control is an important component of malaria control programmes, and it includes Indoor Residual Spraying (IRS) and the use of Long-Lasting Insecticidal Nets (LLINs) [3]. During the early 20th century, IRS was the key malaria control intervention achieving spectacular reduction in malaria [4–6]. In IRS, the insecticide is sprayed on the walls of houses and as a result when mosquitoes rest on these sprayed walls, they pick up the particles of insecticide, and lose their longevity [7]. The same concept applies in LLINs, and in addition, the nets prevent human to mosquito contact.

The World Health Organization (WHO) and Roll Back Malaria Partnership to End Malaria (RBM) have prepared materials and strategies for a malaria-free world by 2030 [8]. SEAR countries identified 2030 as a target for malaria free Asia Pacific. The Indian Government along with other Governments of Asia Pacific countries have committed to this target [9]. National Vector Borne Disease Control Programme (NVBDCP) has launched a national framework to eliminate malaria by 2030 [10]. The Operational Guidelines for Implementation of Malaria Programme 2009 by NVBDCP are followed by the Government of MP [11].

As per the national guidelines, the Government of Madhya Pradesh (GoMP) uses IRS in sub-centres which score 2 to 4.99 on the Annual Parasite Incidence (API). In Mandla, as per Memorandum of Understanding between the Government of MP, Indian Council for Medical Research (ICMR), and Foundation for Disease Control and Elimination of India (FDEC India), this criteria for IRS was revised to 1 to 4.99 to cover more sub-centres. The alpha-cypermethrin (ACM) 5 % insecticide (synthetic pyrethroids) was sprayed with the help of 18 squads consisting of one senior field worker (SFW) and 5 field workers (FW) with two pumps in each squad.

LLIN is a long-term anti-mosquitoes measure with effects lasting for 3–5 years [12]. A meta-analysis of the efficacy of insecticide-treated bed nets in 29 countries of sub-Saharan Africa showed a pooled reduction by 24 % in parasitaemia prevalence in children and 23 % reduction in under five child mortality rates over the last decade [13]. This vector control strategy is also endorsed by the WHO as one of the strategies for controlling and preventing human-to-mosquito contact [12].

## Methods

### Study area

The study was carried out in Mandla district of Madhya Pradesh, which is located in the south-eastern part of the state as part of the Malaria Elimination Demonstration Project [14]. The area is covered with dense forests, valleys and mountains (23° N latitude, 80° 10’ E longitude). The total district area is 8771 km ^2^ and is home to approximately 1.15 million population [15] with about 90 % population living in the rural areas. Mandla experiences summers from March to June, monsoons from July to October, and winters from November to February.

### Study design, training, sampling technique and sample size

In this study, training and supervisory support was provided for both IRS and LLIN campaigns. In the year 2017, IRS was monitored for both rounds consisting of 45 days each. Based upon the findings of 2017, in the year 2018, the supportive supervision of spraying teams along with post-distribution monitoring of usage of LLINs was performed. Based upon the findings of 2018, in the year 2019, supportive supervision for distribution of the LLINs was provided and their usage was observed in 2020.

One-day training was provided to each team on the do’s and don’ts about the IRS and LLINs. The training regimens included components on preparedness, operations, safety measures and evaluation. The spraying team was advised to follow: (1) date-wise route chart; (2) record the number of rooms, houses sprayed, locked or refused in the register; (3) record attendance of the team members; (4) maintain stock register for entry of spray pumps, nozzles, and solution; and (5) maintain the daily diary. Proper training for maintenance of the records was given before IRS programme each year.

During the spray operations, the spray team and supervisor were advised to: (1) check the spray equipment for proper discharge rate; (2) solution preparation; (3) correct mixing; (4) spray technique; and (5) usage of proper safety equipment. They were also trained for post-spray activities i.e. (1) washing of spray equipment; (2) disposal of leftover solution; (3) coverage of rooms; and (4) observation of any side effects. For LLIN distribution and evaluation, the staff was trained to: (1) perform household survey to determine number of required nets; (2) verify distributed vs. required nets; (3) assess proper usage of nets; (4) assess washing practices; and (5) observe any side effects because of usage of nets.

The IRS campaigns were observed during 45 days for each round of spraying (16th June to 31st July and 1st September to 15th October 2017) using a pre-tested, closed-ended monitoring checklist. The checklist consisted of two parts: (1) observations of spraying team; and (2) observations of sprayed houses. Each spraying team used an advance tour plan for spraying of households. The teams were intercepted in their target villages on different days and the questionnaire tool was administered. Each spraying team was monitored using the checklist for 159 times in 2017 and 183 times in 2018. The project staff supervised the IRS operations in 2018. For observing the sprayed houses, one checklist was administered in randomly selected sprayed households yielding a total sample size of 1261 and 1791 in the years 2017 and 2018, respectively.

While the WHO universal coverage proposes use of one LLIN per 1.8 individuals [13]. The NVBDCP has recommended one LLIN should be distributed per 2.5 individuals [16]. Accordingly, the State of MP used NVBDCP recommendation of one net per 2.5 individual. During this study, the use of LLINs was assessed periodically to determine proper usage that interrupts human mosquito contacts. In this study, usage of 4,879 LLINs was checked in about 2000 (5 %) households in 2019 for the nets distributed in 2018. In 2019, MEDP provided supportive supervision to the state government in distribution of next batch of LLINs and checked usage of 4,967 LLINs in about same number of households in 2020.

### Data management and analysis

The data was entered in data entry software designed on CS-Pro 7.0 platform and data analysis was done with Statistical Package for Social Sciences (SPSS) v20.0 by IBM.

## Results

### Indoor Residual Spraying

**Fig. 1:**
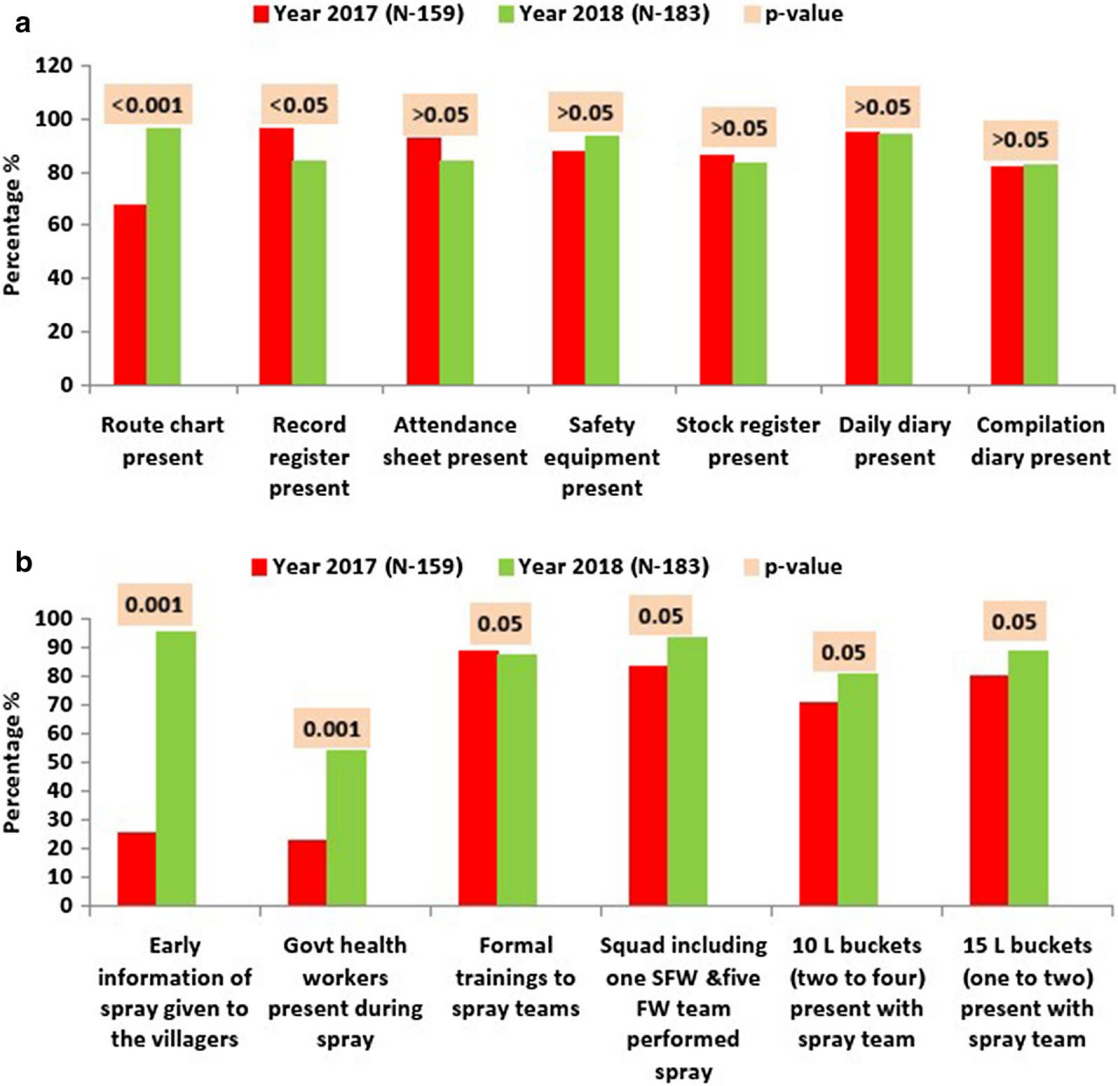
Preparedness activities during IRS 2017 and 2018; **a** Record maintenance, **b **Training, availability of equipment, advance information to villagers, and team composition

The observations of spraying teams were divided into (1) Spraying preparedness; (2) Operation of spraying; and (3) Completion of spraying. The comparison of results obtained for IRS campaigns for 2017 and 2018 are shown in Figs. 1a, b (spraying preparedness), 2a–c (operation of spraying) and 3a, b (safety procedures). The post-spraying observations of households are presented in Table 1. These observations include quality of spraying on the walls, coverage of sprayed (house area), and adverse effects as reported by the household members.

**Table 1:** Post spray observation using questionnaire-based format in 2017 and 2018

Parameters	Year 2017		Year 2018		p-value
	Nos	%	Nos	%	
Household observation					
No of house hold covered	1261		1791		
Has your home been sprayed?	1234	97.8	1778	99.3	< 0.01
Were you informed about the spray in-advance?	272	21.5	1707	95.3	< 0.01
Were you asked to cover the essential equipment before the spray?	1028	81.5	1637	91.4	< 0.01
Were you told not to paint the walls after the spray?	526	41.7	1216	67.9	< 0.01
Spray quality	603	47.8	1586	88.6	< 0.01
Are you satisfied with the spray?	842	66.8	1622	90.5	
Room Observation					
Total targeted rooms for spray	8288		8420		
Completed rooms	5990	72.3	6100	72.4	> 0.05
Partially sprayed	744	8.9	757	8.9	> 0.05
Refused rooms	943	12.4	952	11.3	< 0.05
Locked rooms	239	2.9	239	2.8	> 0.05
Left rooms	372	4.5	372	4.0	> 0.05
Adverse effects noted by the respondents	206	16.3	144	8.0	< 0.05
Adverse effect observation					
Burning on face	69	33.5	35	24.3	> 0.05
Cough	11	5.3	6	4.2	> 0.05
Cold	5	2.0	11	7.6	< 0.05
Dizziness	1	0.5	2	1.4	< 0.05
Eye irritation	31	15.0	13	9.0	> 0.05
Headache	9	4.4	5	3.5	> 0.05
Insomnia	4	1.9	1	0.7	> 0.05
Itching	68	33.0	63	43.8	< 0.05
Runny nose	8	3.9	2	1.4	> 0.05
Stomachache	0	0.0	5	3.5	< 0.01

In the year 2017, it was observed that the spraying teams did not have a route chart with them for spraying in about 33 % of households (Fig. 1a). This advanced route chart comprised of the schedule and route for the IRS that was to be done on a given day. In comparison, in 2018, after monitoring and supervisory support exercise, the advance route chart was present for almost all household sprayed (96.1 %), which was a significant improvement (p < 0.001).

Pre-spraying information to the villagers jumped significantly from 25.2 to 95.2 %. Significant improvement was observed in the attendance of government health workers, who were assigned duties with the spraying teams from 22.6 to 54.3 %. The desired team composition for the IRS spraying (1 Senior Field Worker (SFW) + 5 Field Workers (FW)) was 83.4 % in 2017 and 93.3 % in 2018. As per the guidelines, each team should have two to four 10-litre buckets and one to two 15-litre buckets, which were present 71 % and 79.8 % in 2017. With supportive supervision in 2018, presence of the buckets rose to 80.6 % (10-litre bucket) and 88.7 % (15-litre bucket), respectively (Fig. 1b).

**Fig. 2:**
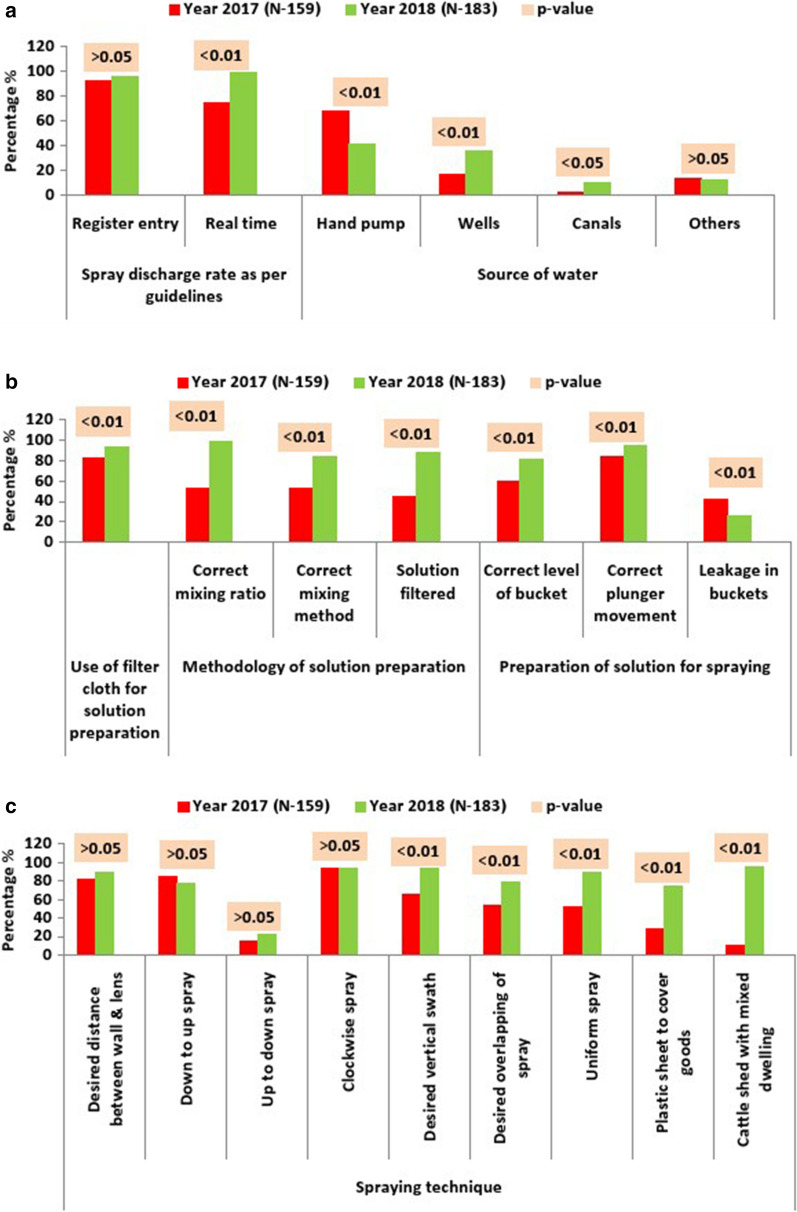
Operational observations carried during IRS 2017 and 2018; **a** Spray discharge rate and source of water, **b** Different components in the methodology of preparation spray solution, **c **Different components in spraying technique

The standard discharge rate for stirrup pumps is 740 to 850 ml per minute. As per the normal practice of spraying teams (each team has two pumps), the discharge rate was recorded during spraying of households which was around 93 % in 2017 and 96 % in 2018, however, it was revealed that the actual discharge rate was 75 % in 2017, which improved to 93 % in 2018. The teams had filtering cloth about 83 % in 2017 which improved to about 94 % in 2018 (Fig. 2a).

Regarding the methodology of preparation of solution for the spraying in 2017, it was observed that the insecticide solution was prepared with 250 gm insecticide in 10 litre water only half of the times (53.7 %). We also found that the solution was mixed properly only 53.6 % of the times and was filtered with a filter cloth only 44.4 % of the times. In 2018, the numbers improved to 98.9 %, 83.3 and 87.4 % for each activity, respectively (Fig. 2b).

For the preparation for spraying (Fig. 2b), it was observed that solution was being filled in the bucket to the desired (two-third) level 60.4 % of the times in 2017 and 81.9 % in 2018. In 2017, the movement of the plunger was between under optimal range (4 to 6 inches) for 84.3 % of the times and leakage was observed in 42 % of the buckets. With supportive supervision, the plunger movement improved to 94.6 % and leakage decreased to 25.6 % (p < 0.01).

**Fig. 3:**
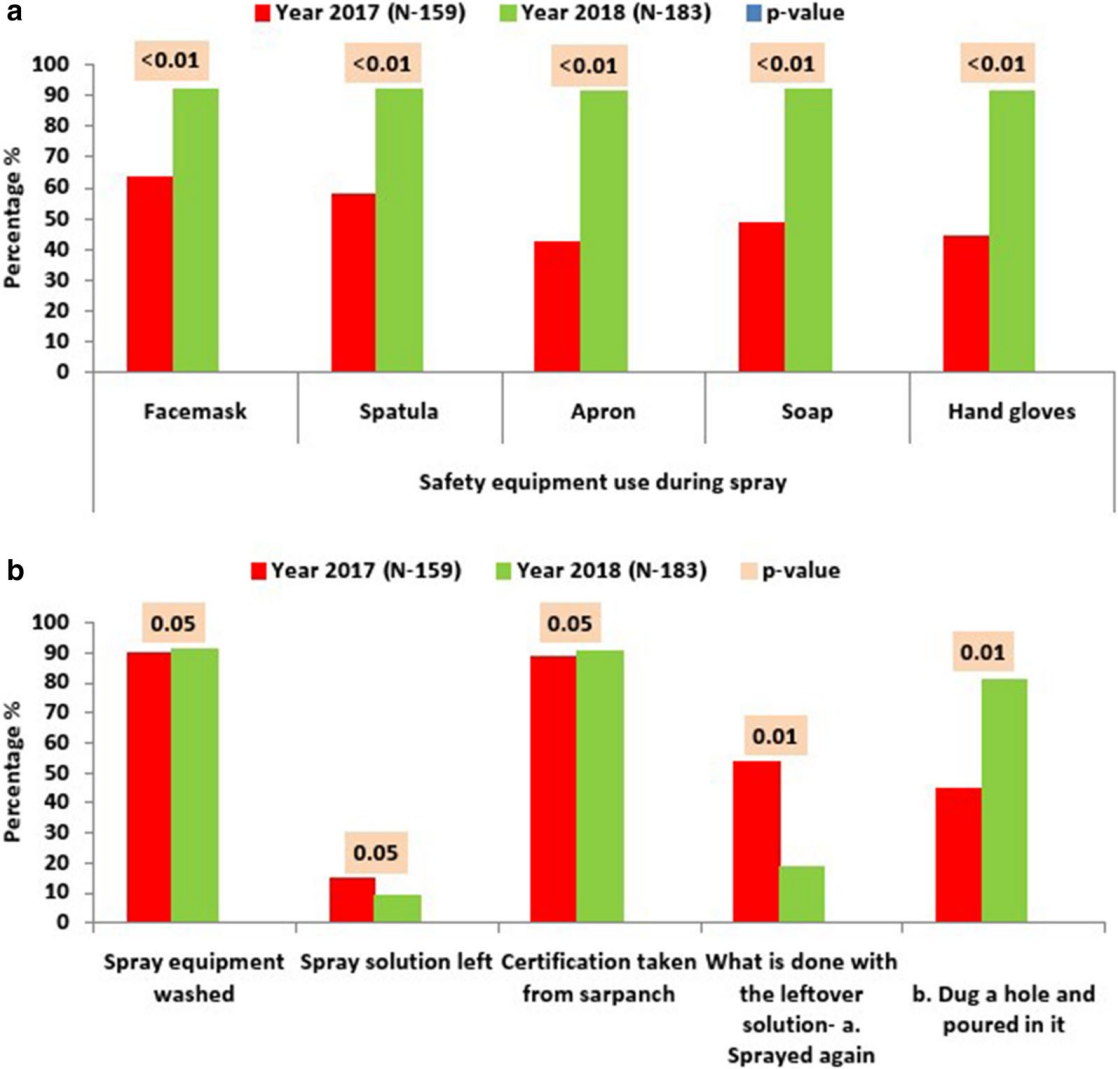
Safety measures and post-IRS observations; **a** Availability of safety measures, **b **Post-spray activities

The technique of spraying (Fig. 2c) observed by the team included distance between wall and lens, spraying direction, uniform or patchy spraying. The desired distance of wall from lens must be generally 1.5 to 2.5 feet, which was achieved 88.7 % of the times in 2018 as compared to 81.8 % in 2017. In the years 2017 and 2018 the clockwise spraying was done 94.3 % and 93 % of times, vertical swath of 75 cm was achieved 65.4 % and 93 % of the times, desired overlapping of spraying was achieved 53.5 % and 78.8 % of the times, down-to-up (correct) spraying was found 84.9 % and 77.9 % of the times and up-to-down spraying (in-correct) was 22.0 % and 15.1 % of the times, respectively. Uniform spraying was achieved only 52.2 % of the times in 2017, whereas in 2018, it improved significantly to 89.9 %. Plastic sheet was used to cover the household items only 28.3 % of the instances in 2017 as compared to 73.6 % in 2018. Spraying on cattle-sheds was done only 10 % of times in 2017. In 2018, this number rose to 95.3 %. When the presence of the safety equipment was physically verified, it was observed that face masks, spatulas, aprons, hand gloves and soaps were present 63.5 %, 58 %, 42.1 %, 44 % and 48.4 % of the times and 92.1 %, 92.1 %, 91.5 %, 91.5 % and 92.1 % in the years 2017 and 2018, respectively (Fig. 3a). All these improvements were statistically significant.

After the completion of the spraying, the leftover solution was used again for spraying in 45 % of the cases and disposed by pouring the solution in a hole dug in the ground 54 % of the times, whereas these numbers changed into 18.9 % and 81.1 % after supervision of 2018 (p < 0.05) (Fig 3b).

### Post spraying observations

A total of 1261 in 2017 and 1791 in 2018 households were assessed for awareness of community for spraying, its quality, coverage and adverse effects (Table 1). A total of 97.8 % and 99.3 % households 2017 and 2018, respectively, informed that their houses were sprayed (p < 0.01). In 2017, 21.5 % households were informed about the spraying in-advance, but in 2018, the number increased significantly (95.31 %). When the respondents were asked to cover the household items before the spraying, the number rose from 81.5 to 91.4 %. The number of households asked not to paint the walls after the spraying also increased from 41.7 % to 2017 to 67.9 % in 2018. The spraying quality improved from 47.8 % to 2017 to 88.6 % in 2018. Improvement in spraying satisfaction by the respondents increased significantly to 90.5 % in 2018 from 66.8 % to 2017 (Table 1).

A total of 8,288 and 8,420 targeted rooms in 2017 and 2018, respectively, were checked for spraying (Table 1). About 16 % and 8 % households complained about adverse effects of the insecticide sprayed in the year 2017 and 2018, respectively. The most common adverse effects included face burning, itching, eye irritation.

### Long‐Lasting Insecticidal Nets

In 2017, a total of 124,726 LLINs were distributed in 319 study villages in 61 sub-centres in eight blocks, and in 2019, the second round of LLINs (250,250) were distributed in 459 study villages in 95 sub-centres in nine blocks of Mandla district. The door-to-door surveys of sample households was conducted in the year 2019 where nets were distributed in 2017 and in the year 2020 where nets were distributed in 2019, using the questionnaire that captured information related to bed-net distribution, acceptance, usage, awareness of community, side effects etc. (Table 2). These surveys were carried out after six to eight months of distribution of LLINs in 2017 and 2019. Based on the findings of the 2017 assessment, the supportive supervision was provided for distribution of LLINs in 2019. In 2019 survey, almost all (99.6 %) bed-nets were provided for use to the villagers. The usage of LLINs in two categories i.e. regular usage (nets being used by the residents throughout the year) and occasional usage (nets being used by the residents sporadically during high transmission seasons), was assessed. Only 34 % nets were found being used regularly. The rest of them used occasionally, mainly during the monsoon season. Whereas in 2019, the regular usage rose to 47 % (p < 0.01).

**Table 2:** Household survey for LLIN assessment

Parameters	N (%) 2017–2018	N (%) 2019–2020	p-value
Households (HH) surveyed	2000	2000	–
Population surveyed	12,200	9683	–
Number of LLINs distributed in the surveyed households	4879	4967	–
Number of nets being used by the members regularly	1638 (33.7 %)	1991 (47.4 %)	< 0.01
Number of nets being used by the members occasionally	3231 (66.3 %)	2207 (52.6 %)	< 0.01
Demonstration received by HH during distribution	41.6 %	94.4 %	< 0.01
Percent of HH spending night outside	7.0 %	1.6 %	
Percent of HH who took LLINs when spent night outside the house	33.3 %	75 %	< 0.01
Percent of HH actually used net who took LLINs when spent night outside	28.1 %	100 %	< 0.01
Percent of HH used domestic methods apart from LLINs	73.1 %	82.8 %	< 0.01
Percent of HH experienced any adverse effect	26.7 %	9.6 %	< 0.01
Itching	91 %	88.5 %	
Sleeplessness	2 %	0.5 %	
Eye irritation	6 %	9 %	
Headache	1 %	1 %	
Percent of HH received IEC/BCC messages or LLIN usage demos during distribution	3.0 %	89.4 %	< 0.01

In 2017, out of the total population that received LLINs, 75.70 % and 22.50 % belonged to Schedule Tribes (ST) and Other Backward Castes (OBC), respectively. Whereas, only 1.40 % and 0.50 % belonged to Scheduled Caste (SC) and General categories, respectively. Majority of heads of the families are farmers (68 %) followed by 27 % daily wage labourers. Other professions constituted less than 1 % of the total population. These statistics were similar in 2019 as well. In 2017, LLINs were received by 98.1 % of the families. Out of which, 97.6 % people responded that they received bed-nets from the health department, 1.9 % said that they do not know who gave them the bed nets and 0.4 % said they got it from the market. This pattern of LLIN distribution also remained similar in the 2020. On an average, two LLINs have been received by each household and same number was available during the study. In each household, an average of four people used the LLINs on a daily basis.

In 2017, demonstration on the correct usage of LLINs was received by 41.6 % recipients. There was significant diversity in the demonstration of usage provided to the villagers of the district. In the Niwas block 83.4 % of villagers stated receiving in demonstrations, while Mohgaon and Bicchiya, 33 % and 25.4 % received demonstrations, respectively. In the year 2019, the figures jumped up-to 94.4 % (p < 0.01) with no significant difference between individual blocks.

In 2017, out of the 7 % people who reported spending nights outside their homes in the last week, majority (66.7 %) reported not taking the LLINs with them (Table 2). However, in 2019, lesser people reported sleeping outside their home (1.6 %), but higher percentage (75 %) of people carried the LLINs with them (p < 0.01). Out of the remaining people who took the LLINs outside, only 28 % used them in 2017 and everyone reported using them in 2019 (100 %) (p < 0.01).

Almost all the respondents in 2017 knew the purpose of LLINs provided to them (99.4 %) Amongst all blocks, Bicchiya showed least acceptance to the LLINs (81 %), rest of the blocks showed an acceptance of 90 % or higher with an average of 91 % acceptance. However, no significant variation between individual blocks was seen in 2019.

In 2017, study revealed that 73.1 % of villagers used other domestic methods for protection against mosquitoes, and this number further rose to 82.8 % in 2019 (Table 2) Most of the residents that used alternative forms of protection were from Ghughari and least were from Narayanganj. About 90 % of villagers reported using smoke from paddy, dried cow dung, neem, wood and garbage as the preferred way of prevention against mosquitoes. Few more options like use of fans, electric racquets etc. were reported by 8.37 % people. A small amount of people also use incense sticks (0.64 %), synthetic repellents like vaporisers (0.86 %) and only 0.21 % people believed that good sanitation and hygiene helps in prevention.

A total of 71.4 % people reported no suffocation while sleeping inside the bed nets and 97.7 % people used it correctly (by hanging it). Majority of people (92.7 %) also reported reduction in mosquito bites by using these bed nets. Almost three-fourth respondents (73.31 %) did not experience any adverse effects by these nets in 2017. This number increased to 90 % in 2019 (p < 0.01). Those who suffered from adverse effects, 91 % had itching, 5.35 % eye irritation, 2 % sleeplessness and 1.26 % headache in 2017 with similar proportions in 2019 (Table 2).

In 2017, almost all (97 %) respondents informed that no awareness campaigns were conducted during or after distribution of LLINs by the state government. The situation was reversed in 2019, with almost 90 % respondents (p < 0.01) confirming the awareness campaigns conducted for the community (Table 2).

## Discussion

There was significant improvement in the outcome of IRS as a result of supportive supervision between 2017 and 2018. In the year 2017, between the spraying dates of 16th June to 31st August and 16th September to 30th October, a total of 301 malaria cases were detected in the district, whereas, within the same dates in the year 2018, only 43 malaria cases were found. This significant drop may be attributed in-part due to the improvement in spraying quality during 2018. Additionally, entomological investigations as part of this project [17] has revealed 41 % mortality of *Anopheles culicifacies* on 30-day post-spraying in July 2017 and 61.3 % in July 2018. This can also be attributed to the improved quality of spraying in 2018. However, there was a reduced mortality (44.4 %) of *An. culicifacies* in October 2018 [17] which suggests the need for stringent training, monitoring, and supervision during IRS campaigns [18].

It was noticed that spraying teams behave and operate much more efficiently in the presence of a supervisor. The art of spraying which involves movement of hands in defined direction and particular order is critical for the success of IRS campaigns. Therefore, the business-as-usual practice by spraying teams that are not provided routine trainings and are not subjected to in-person supervision most likely results in ineffective spraying. These observations inform us that the spraying teams should have full-time supervision and be regularly trained so that investments in IRS produce the desired result.

An earlier study conducted in district Betul of Madhya Pradesh, malaria was controlled by using IRS along with other intervention during the year 2001 to 2005. In this study, the slide positivity rate (SPR) in pre-intervention stage was > 47 % which declined gradually to 1.3 % [19]. Another key observation from this study was that the vector density also declined significantly after intervention [19]. The present study has revealed about 91 % reduction of indigenous cases of malaria during the period from June 2017 to May 2020, through case management and vector control strategies [20]. Another study of malaria control was conducted in the *Baigachak* area of Dindori district of Madhya Pradesh, where a remarkable 89 % reduction in malaria prevalence was achieved in the year 2013-14 using combined intervention measures IRS, LLINs, prompt diagnosis and treatment along with intensive Information, Education and Communication (IEC) [21].

IRS and LLINs are effective for limiting malaria transmission [3]. The results from randomized comparisons of IRS *versus* no IRS revealed that IRS reduces malaria incidence in unstable malaria settings [22]. Similar findings were found in Equatorial Guinea and Western Kenya [23, 24]. Similarly, malaria control programmes in Europe, Asia and the Americas that have resulted in saving hundreds of millions of lives between the 1940s and the 1980s, benefitted by IRS as a vector-control intervention [25].

Based on the findings, it is suggested that IRS operations, while they may seem simple, but in practice, requires management by skilled professional staff. The supervisor as well as the spraying team has to understand local epidemiology, transmission dynamics, vector behaviour and insecticide resistance status [26]. It is worth noting that similar observations were also made in a study of IRS usage in Visceral Leishmaniasis control [27].

In the LLIN component of the present study, the average acceptance of use of LLINs was observed 91 %. These findings are different from an assessment study done in the Buie and Fentalie districts of Ethiopia, where the rate of retention of bed nets was 72 %, and 60.5 %, respectively. The reason for difference of Ethiopian study with our study towards acceptance or retention may be due to the fact that our assessment was done after six to eight months of distribution of LLINs, whereas, in Ethiopia the difference between distribution and assessment was three years. In the same study, majority of people knew that the nets were for protection against mosquito bites (82.4 %) and malaria (14.3 %) [28], which is similar to the findings of the present study where 99.4 % were aware about the purpose of bed nets.

There was also an increase in number nets being used regularly by the residents in 2019-20 as compared to 2017–2018. This increase in usage of beds nets can be attributed to awareness campaigns that informed the villagers about benefits of bed nets as well as their proper use. During the distribution in 2019, MEDP stationed its staff at each LLIN distribution site in the district and facilitated the process along with live demonstrations and IEC/BCC messages to each recipient. This helped in increasing usage of LLINs, acceptance of adverse effects, and increase in usage of nets while traveling outside homes.

Additionally, using supportive supervision, strict monitoring was done to ensure fair distribution practices, which has yielded a near zero complaints of money charged for LLIN distribution (0.2 %) as compared to 2017 (3.8 %). In 2019-2020, lesser number of people reported spending nights outside their homes. This may be due to the peak winters at the time of the survey. However, more people took LLINs with them for spending nights as compared to the 2017 survey. This improvement can be attributed to regular inter-personal communication conducted by MEDP during regular field visits to these villagers.

A systematic review of the Cochrane database to compare ITN vs. curtains or no bed-nets, has revealed that ITNs provide 17 % more efficacy compared to no nets, which results in 5.5 lives saved each year for every 1000 children protected with ITNs. In areas of stable malaria, ITNs reduced the incidence of uncomplicated malaria by 50 % and in unstable areas by 62 % when compared to no nets. ITNs also had impact on severe malaria, parasite prevalence, high parasitaemia, splenomegaly and also improved average haemoglobin level in children by 1.7 % packed cell volume [29].

An evaluation of LLIN distribution in Togo, West Africa, was done, where 93.4 % respondents received visits and awareness messages from a community health worker [30]. In the present study, almost none of the respondents received any awareness drives by the health worker on proper usage of these nets in 2017. Therefore, awareness campaigns should be conducted during distribution of LLINs in addition, strict monitoring and follow-up after distribution of LLINs should be built-in as a practice.

After supervision and monitoring in 2019, our study showed improvement in awareness by over 97 %, which is better than the West Africa evaluation [30]. In Zambia, Masaninga et al. concluded that door to door delivery of LLINs ensured availability of nets in hard-to-reach areas and provided a good opportunity to educate the members face-to-face. IEC/BCC activities during and after distribution played a key role in increasing compliance of LLIN usage amongst the communities [31]. In this study, the mode of distribution of nets from one focal point in one village could not be changed to door-to-door delivery. However, it was ensured that the visits of Village Malaria Workers raised awareness using inter-personal communication techniques resulting in better usage of LLINs.

Regarding the reported adverse-effects of the bed-nets, a community-based survey in Uttar Pradesh found that 2.8 % of respondents reported itching and irritation by using the bed nets [32]. In this study, complaints on adverse effects reduced from 26.7 to 9.6 % between 2017 and 2019, which is a reduction by 64 %. The significant difference can be attributed to use of IEC/BCC during the 2019, which informed the recipients about common expectations from the bed-nets in terms of adverse-effects and proper washing and drying techniques. It was noted that if the recipients are better informed about the LLINs, they exhibit better compliance.

Sood et al. found in Uttar Pradesh that use of smoke using similar methods was much more in villages where LLINs were not distributed as compared to those where LLINs were distributed [32]. However, in the present study it was noticed that significant use of smoke was done as an alternative to LLINs. When asked the reason, respondents argued that LLINs only protect them during sleeping but to enjoy their evening time, they use smoke to ward off the mosquitoes. IRS and LLINs are equally effective interventions because study area had higher proportion *An. culicifacies*, which is known to be an endophilic species [17] and malaria transmission is maintained by this species from July to October.

## Conclusions

This study provides evidence that effective supervision and training improves the quality of IRS and usage of LLINs. Supervision included active monitoring of each step of the spraying operations followed by post-spraying assessment of the sprayed households. For LLIN, IEC/BCC campaigns were conducted at each distribution site with live demonstrations of hanging the bed-nets followed by a regular door-to-door visits and conduction of awareness drives through inter-personal communication. Based on the results of this study, It is suggested that continued training and monitoring of staff that is deployed to spray houses and distribute bed nets. The study also revealed that proper IEC/BCC drives help increase community acceptance of vector control measures and their rational usage.

## Data Availability

We have reported all the findings in this manuscript. The hardcopy data is stored at MEDP Office in Mandla, Madhya Pradesh and National Institute of Research in Tribal Health (NIRTH), Indian Council of Medical Research (ICMR), Jabalpur, Madhya Pradesh. Softcopy data is available on the project server of MEDP hosted by Microsoft Azure. If anyone wants to review or use the data, they should contact.

## Abbreviations

ACM: Alpha-Cypermethrin
API: Annual Parasite Incidence
FDEC: Foundation for Disease Control and Elimination
FW: Field Worker
GoMP: Government of Madhya Pradesh
ICMR: Indian Council of Medical Research
IEC: Information, Education and Communication
IRS: Indoor Residual Spraying
LLIN: Long-Lasting Insecticidal Nets
MEDP: Malaria Elimination Demonstration Project
MP: Madhya Pradesh
NVBDCP: National Vector Borne Disease Control Programme
OBC: Other Backward Castes
RBM: Roll Back Malaria Partnership to End Malaria
SC: Scheduled Castes
SEARO: South-East Asia Region
SFW: Senior Field Worker
SPR: Slide Positivity Rate
ST: Scheduled Tribes
WHO: World Health Organization

## References

[1] WHO SEARO. Anopheline species complexes in South and South-east Asia. World Health Organization Regional Office for South East Asia, 2007.

[2] Directorate of Health Services. Ministry of Health and Family Welfare. Malaria situation in India. National Vector Borne Disease Control Programme, New Delhi, 2019 [Available from: https://nvbdcp.gov.in/index4.php?lang=1&level=0&linkid=564&lid=3867.

[3] Raghavendra K, Barik TK, Reddy BN, Sharma P, Dash AP (2011). Malaria vector control: from past to future. Parasitol Res.

[4] Shiff C (2002). Integrated approach to malaria control. Clin Microbiol Rev.

[5] Mabaso ML, Sharp B, Lengeler C (2004). Historical review of malarial control in southern African with emphasis on the use of indoor residual house-spraying. Trop Med Int Health.

[6] Wakabi W (2007). Africa counts greater successes against malaria. Lancet.

[7] National Vector Borne Disease Control Programme. Guidelines for indoor residual spraying. New Delhi. 2018 [Available from: https://www.nvbdcp.gov.in/WriteReadData/l892s/Indoor%20Residual%20Spray%20-%205.doc.

[8] RBM. Malaria - toolkits. Geneva, RBM Partnership to end malaria. [Available from: https://endmalaria.org/resources/library?type=3.

[9] Asia Pacific Leaders Malaria Alliance. APLMA roadmap for malaria elimination. [Available from: https://www.aplma.org/upload/resource/files/APLMA_Roadmap_2017.pdf.

[10] National Vector Borne Disease Control Programme. National Framework for Elimination of Malaria in India 2016-30. New Delhi, 2015.

[11] National Vector Borne Disease Control Programme. Operational manual for implementation of malaria programme. Ministry of Health and Family Welfare; New Delhi, 2009 Available from: https://nvbdcp.gov.in/Doc/Malaria-Operational-Manual-2009.pdf.

[12] WHO. Malaria - Key Facts. Geneva. [Available from: https://www.who.int/news-room/fact-sheets/detail/malaria].

[13] WHO. Revised recommendations for achieving universal coverage with long-lasting insecticidal nets in malaria control. Geneva: Malaria Policy Advisory Group Meeting, Global Malaria Programme, World Health Organization. 2017. [Available from: https://www.who.int/malaria/mpac/mpac-oct2017-erg-universal-coverage-session9-presentation.pdf?ua=1].

[14] Rajvanshi H, Bharti PK, Nisar S, Jain Y, Jayswar H, Mishra AK (2020). Study design and operational framework for a community-based Malaria Elimination Demonstration Project (MEDP) in 1233 villages of district Mandla, Madhya Pradesh. Malar J.

[15] Sharma RK, Rajvanshi H, Bharti PK, Nisar S, Jayswar H, Mishra AK (2021). Socio-economic determinants of households in tribal dominated Mandla district enrolled in Malaria Elimination Demonstration Project in Madhya Pradesh. Malar J.

[16] National Vector Borne Disease Control Programme. Guideline on distribution and effective use of long lasting insecticidal nets (LLIN). Ministry of Health and Family Welfare; New Delhi, 2009 [Available from: https://www.nvbdcp.gov.in/WriteReadData/l892s/guidline-supply-distribution-comm-LLIN-orissa.pdf.

[17] Mishra AK, Bharti PK, Vishwakarma A (2020). A study of malaria vector surveillance as part of the Malaria Elimination Demonstration Project in Mandla. Madhya Pradesh. Malar J.

[18] Rajvanshi H, Nisar S, Bharti PK, Jayswar H, Mishra AK, Sharma RK (2021). Significance of training, monitoring and assessment of malaria workers in achieving malaria elimination goal of Malaria Elimination Demonstration Project. Malar J.

[19] Singh N, Shukla M, Mishra A, Singh M, Paliwal J, Dash A (2006). Malaria control using indoor residual spraying and larvivorous fish: a case study in Betul, central India. Trop Med Int Health.

[20] Bharti PK, Rajvanshi H, Nisar S, Jayswar H, Saha KB, Shukla MM (2020). Demonstration of indigenous malaria elimination through Track-Test-Treat-Track (T4) strategy in a Malaria Elimination Demonstration Project in Mandla, Madhya Pradesh. Malar J.

[21] Singh N, Mishra AK, Saha KB, Bharti PK, Sisodia DS, Sonal GS (2018). Malaria control in a tribal area of central India using existing tools. Acta Trop.

[22] Pluess B, Tanser FC, Lengeler C, Sharp BL (2010). Indoor residual spraying for preventing malaria. Cochrane Database Syst Rev.

[23] Sharp BL, Ridl FC, Govender D, Kuklinski J, Kleinschmidt I (2007). Malaria vector control by indoor residual insecticide spraying on the tropical island of Bioko, Equatorial Guinea. Malar J.

[24] Zhou G, Githeko AK, Minakawa N, Yan G (2010). Community-wide benefits of targeted indoor residual spray for malaria control in the western Kenya highland. Malar J.

[25] WHO. Indoor residual spraying: an operational manual for indoor residual spraying (IRS) for malaria transmission control and elimination. Geneva, World Health Organization, 2015.

[26] WHO (2006). Indoor residual spraying: use of indoor residual spraying for scaling up global malaria control and elimination.

[27] Huda MM, Mondal D, Kumar V, Das P, Sharma S, Das ML (2011). Toolkit for monitoring and evaluation of indoor residual spraying for visceral leishmaniasis control in the Indian subcontinent: application and results. J Trop Med.

[28] Fettene M, Balkew M, Gimblet C (2009). Utilization, retention and bio-efficacy studies of PermaNet® in selected villages in Buie and Fentalie districts of Ethiopia. Malar J.

[29] Lengeler C (2004). Insecticide-treated bed nets and curtains for preventing malaria. Cochrane Database Syst Rev.

[30] Stevens ER, Aldridge A, Degbey Y, Pignandi A, Dorkenoo MA, Hugelen-Padin J (2013). Evaluation of the 2011 long-lasting, insecticide-treated net distribution for universal coverage in Togo. Malar J.

[31] Masaninga F, Mukumbuta N, Ndhlovu K, Hamainza B, Wamulume P, Chanda E (2018). Insecticide-treated nets mass distribution campaign: benefits and lessons in Zambia. Malar J.

[32] Sood RD, Mittal P, Kapoor N, Razdan R, Dua V, Dash A (2010). Community awareness, perceptions, acceptability and preferences for using LLIN against malaria in villages of Uttar Pradesh, India. J Vector Borne Dis.

